# Studying Surface Chemistry of Mixed Conducting Perovskite Oxide Electrodes with Synchrotron-Based Soft X-rays

**DOI:** 10.1021/acs.jpcc.3c04278

**Published:** 2023-10-09

**Authors:** Zijie Sha, Gwilherm Kerherve, Matthijs A. van Spronsen, George E. Wilson, John A. Kilner, Georg Held, Stephen J. Skinner

**Affiliations:** † Department of Materials, Imperial College London, Exhibition Road, London SW7 2AZ, U.K.; ‡ Diamond Light Source Ltd, Didcot OX11 0DE, U.K.

## Abstract

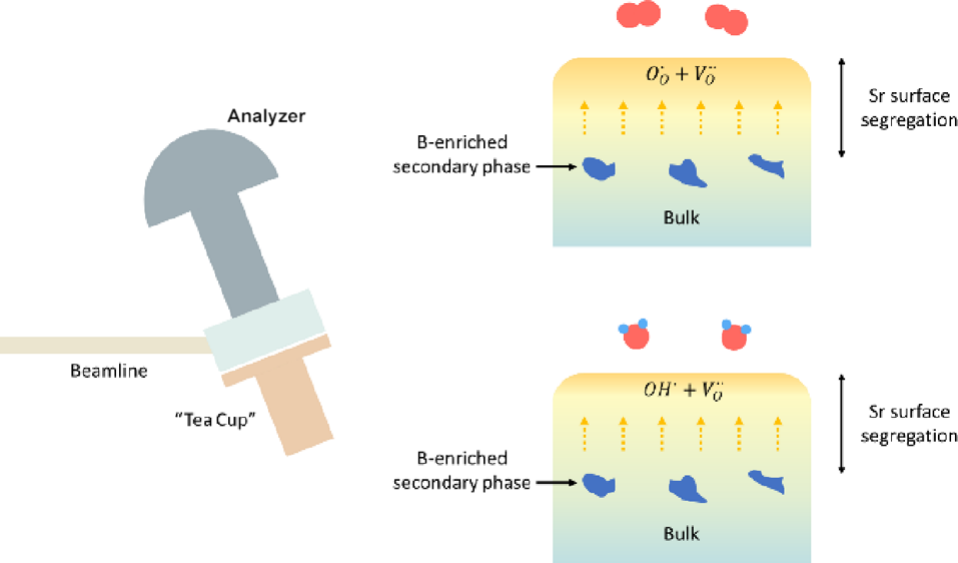

A fundamental understanding of the electrochemical reactions and surface chemistry at the solid–gas interface *in situ* and *operando* is critical for electrode materials applied in electrochemical and catalytic applications. Here, the surface reactions and surface composition of a model of mixed ionic and electronic conducting (MIEC) perovskite oxide, (La_0.8_Sr_0.2_)_0.95_Cr_0.5_Fe_0.5_O_3-δ_ (LSCrF8255), were investigated *in situ* using synchrotron-based near-ambient pressure (AP) X-ray photoelectron spectroscopy (XPS) and near-edge X-ray absorption fine-structure spectroscopy (NEXAFS). The measurements were conducted with a surface temperature of 500 °C under 1 mbar of dry oxygen and water vapor, to reflect the implementation of the materials for oxygen reduction/evolution and H_2_O electrolysis in the applications such as solid oxide fuel cell (SOFC) and electrolyzers. Our direct experimental results demonstrate that, rather than the transition metal (TM) cations, the surface lattice oxygen is the significant redox active species under both dry oxygen and water vapor environments. It was proven that the electron holes formed in dry oxygen have a strong oxygen character. Meanwhile, a relatively higher concentration of surface oxygen vacancies was observed on the sample measured in water vapor. We further showed that in water vapor, the adsorption and dissociation of H_2_O onto the perovskite surface were through forming hydroxyl groups. In addition, the concentration of Sr surface species was found to increase over time in dry oxygen due to Sr surface segregation, with the presence of oxygen holes on the surface serving as an additional driving force. Comparatively, less Sr contents were observed on the sample in water vapor, which could be due to the volatility of Sr(OH)_2_. A secondary phase was also observed, which exhibited an enrichment in B-site cations, particularly in Fe and relatively in Cr, and a deficiency in A-site cation, notably in La and relatively in Sr. The findings and methodology of this study allow for the quantification of surface defect chemistry and surface composition evolution, providing crucial understanding and design guidelines in the electrocatalytic activity and durability of electrodes for efficient conversions of energy and fuels.

## 1 Introduction

In the search for efficient and carbon neutral energy storage and conversion devices to lessen the pressing issues of climate change and energy shortage, focus has been directed toward electrochemical systems such as solid oxide cells (SOCs). These cells, which rely on oxygen ion transport, can either produce power through fuel cell mode, and *vice versa* store energy in chemical form through electrolysis mode. Mixed ionic and electronic conducting (MIEC) perovskite oxides (ABO_3_) are widely applied as electrodes for SOCs due to their well-balanced properties including electrocatalytic activities, ionic and electronic conductivity, affordability, and chemical and redox stability.^1−8^ During operation of SOCs, oxygen is either incorporated into or released from the MIEC electrode across the solid–gas interface. This oxygen surface exchange kinetics plays a crucial role in determining the overall performance of SOCs and has been the focus of many studies and reviews. It has been demonstrated to be strongly influenced by two factors, surface reaction mechanisms and surface chemical compositions.

One of the surface reaction mechanisms involves the surface redox center of oxygen exchange reactions.^9−13^ Suntivich et al.^10,11^ proposed the e_g_ filling of the B-site transition metal (TM) cations in the perovskite oxide can be used as an “activity descriptor” to predict the electrocatalytic activity for oxygen reduction reaction (ORR) and oxygen evolution reaction (OER). This is based on the finding that the overlap between the e_g_ orbital of the TM ions and the O 2p_σ_ orbital is stronger than that between the t_2g_ of the TM ions and the O 2p_π_ orbital. On the other hand, recent *in situ* and *operando* studies have revealed that the surface lattice oxygen is also an important redox partner with the oxygen adsorbate.^12,13^ Another aspect of the mechanisms is regarding the differences between O_2_ and other oxygen bearing molecules such as H_2_O.^14−18^ Typical ORR involves oxygen incorporation into the surface and charge transfer, which is described by eq 1 in Kröger–Vink notation:
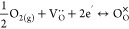
1where V_O_^··^ denotes oxygen vacancies and O_O_^×^ represents a neutral lattice oxygen. Studies have demonstrated an enhanced kinetics for water electrolysis as compared to ORR potentially due to differences in the underlying mechanisms.^14,16,17^

Given that the oxygen exchange reaction is mediated on the electrode surface, it is not surprising that its kinetics are also closely related to the surface’s chemical composition and structure.^19−27^ Oxygen vacancies have been proven to play a crucial role for oxygen transport properties, and the surface oxygen vacancy level has also been proposed as a decisive factor for the oxygen exchange kinetics.^28^ Furthermore, significant compositional and structural deviations have been observed in the near-surface region due to surface instabilities.^25^ In particular, surface segregation and phase separation of the Sr substituent is a known issue that directly impacts the oxygen exchange kinetics and the stability of the electrodes.^15,19−27,29−44^ Sr excess and enrichment have been frequently found on the surface as compared to the bulk composition of the electrode under harsh environments in which they function. The Sr cations can further accumulate and form Sr-enriched phases and surface layers. The key driving forces for Sr segregation have been proposed as the elastic interaction and electrostatic interaction between the host (La^3+^) and substituent (Sr^2+^) cations.^21^ The elastic interaction is due to the size mismatch, and the electrostatic interaction is due to the abundance of oxygen vacancies (V_O_^••^) in the near-surface region of MIEC oxides, which attracts the negatively charged defects defect (Sr_La_^′^) to the surface according to Coulomb’s law. The extent of Sr segregation has been found to correlate with the material’s chemical composition, and external conditions such as temperature, oxygen partial pressure, and electric field.^15,16,21,26,27,29,42,43^ Furthermore, crystal orientation and misorientation features within materials such as grain boundaries and dislocations can also effect on the Sr segregation level.^29,34^

Despite previous extensive studies, the majority of surface characterization studies have been conducted *ex situ*, which only represent “snapshots” of the dynamic surface chemical evolution with defined annealing conditions and time. In addition, the response of the dynamic electrode–gas interfaces to changes in the gas phase is not well understood due to the lack of direct *in situ* experimental evidence. In recent years, synchrotron-based ambient pressure (AP)–X-ray photoelectron spectroscopy (XPS) and near-edge X-ray absorption fine-structure spectroscopy (NEXAFS, also known as X-ray absorption spectroscopy (XAS)) have been developed and demonstrated to be one of the essential techniques for studying electrochemical devices under operating conditions.^44−49^ In this study, the surface defect equilibria and surface composition evolution of (La_0.8_Sr_0.2_)_0.95_Cr_0.5_Fe_0.5_O_3-δ_ (LSCrF8255), a model MIEC perovskite oxide, were investigated using AP-XPS and AP-NEXAFS. The measurements were carried out under dry oxygen and water vapor gas flow, to reflect the implementation of the materials for oxygen reduction/evolution and water electrolysis. LSCrF8255 was chosen due to its excellent bulk stability under both conditions at elevated temperatures.^16^ This work sheds light on the reactivity and durability of perovskite electrode materials in relation to gases and provides guidelines for material design, performance, and durability, not only for SOC technology but also for a wider range of MIEC perovskite oxide applications such as oxygen transport membranes (OTMs) and OTM-based reactors.

## 2 Methods

The A-site deficient (La_0.8_Sr_0.2_)_0.95_Cr_0.5_Fe_0.5_O_3-δ_ (LSCrF8255) powders were supplied by Praxair, Inc. (LOT: 03-P6760DM). Dense ceramic pellets (>97% of the theoretical density) were prepared through uniaxially pressing the powder at a load of 2 t in a 13 mm-diameter die, followed by sintering the pressed green pellets in static laboratory air in a muffle furnace at 1450 °C for 6 h. The sintered pellets were ground with successive grades of SiC papers (Struers Ltd., UK) with grits of 400, 600, 800, and 1200 and then polished with water-based diamond suspensions (Struers Ltd., UK) of 6, 3, 1, and 1/4 μm sequentially to reduce any errors arising from surface roughness.

AP-XPS and NEXAFS measurements were conducted on the B07-C versatile soft X-ray (VerSoX) beamline at Diamond Light Source Ltd., UK.^50^ The LSCrF8255 sample was mounted on a ceramic heater, which was designed and manufactured to fit the “Tea Cup” reaction cell^50^ at the B07-C VerSoX beamline with three stainless-steel clamps. A thermocouple and a gold foil were attached to the top of the sample, as demonstrated in Figure S1 in the Supporting Information (SI). During the measurements, the surface temperature was kept at 500 ± 5 °C using a PID controller and monitored with the thermocouple. There were two reactive gas environments, oxygen gas (99.9992%, H_2_O 0.5 ppm, Air Products) and water vapor. The gas pressure in the experiment chamber was regulated manually to 1 mbar using a mass flow controller for oxygen gas and a leak valve for water vapor. Each sample was initially characterized under ultrahigh vacuum (UHV), and subsequently under each atmosphere. All the AP-XPS spectra and AP-NEXAFS spectra were recorded time-resolved. The AP-XPS spectra were collected with a 20 eV analyzer pass energy and a 600 I/mm grating, along with selected photon energies to ensure the collected photonelectrons had the same kinetic energy (200 eV) and probe depth (approximately 1–1.5 nm). The binding energy (BE) was calibrated using the Au 4f spectra measured on the Au foil. The XPS spectra were processed using the CasaXPS^51^ program, to subtract a Shirley-type^52^ background and fit peaks with a Gaussian–Lorentzian line shape. The AP-NEXAFS spectra were collected in the total electron yield (TEY) mode using the analyzer cone biased at +5 V as electron collector, with a 600 I/mm grating and an estimated information depth of approximately 4–5 nm.^53,54^ The Cr *L*-edge spectra for octahedrally (O_h_) coordinated Cr^3+^ and Cr^4+^ cations were simulated using the charge-transfer multiplet (CTM) approach^55,56^ with the CTM4XAS program.^57^ The simulation parameters are summarized in Table S1 in the Supporting Information, where the ion configuration, crystal field splitting, spin–orbit coupling, and charge transfer effects were considered. Literature values were used as initial guidance.^48,58,59^

Post AP-XPS and AP-NEXAFS, the samples were examined with scanning electron microscopy (SEM) using a LEO Gemini 1525 field emission gun (FEG) SEM (Carl Zeiss AG, Germany) equipped with an INCA X-act energy-dispersive X-ray spectrometry (EDX) detector (Oxford Instruments Ltd., UK). Further investigation into the crystal structure was performed through X-ray diffraction (XRD) analysis with a PANalytical MPD instrument (PANalytical Plc, UK) using a 0.004° step size and a 349.9 s counting time per step. The XRD patterns were fitted with the FullProf^60^ software suite.

## 3 Results and Discussion

### 3.1 Valence States in O_2_ and H_2_O

**Figure 1:**
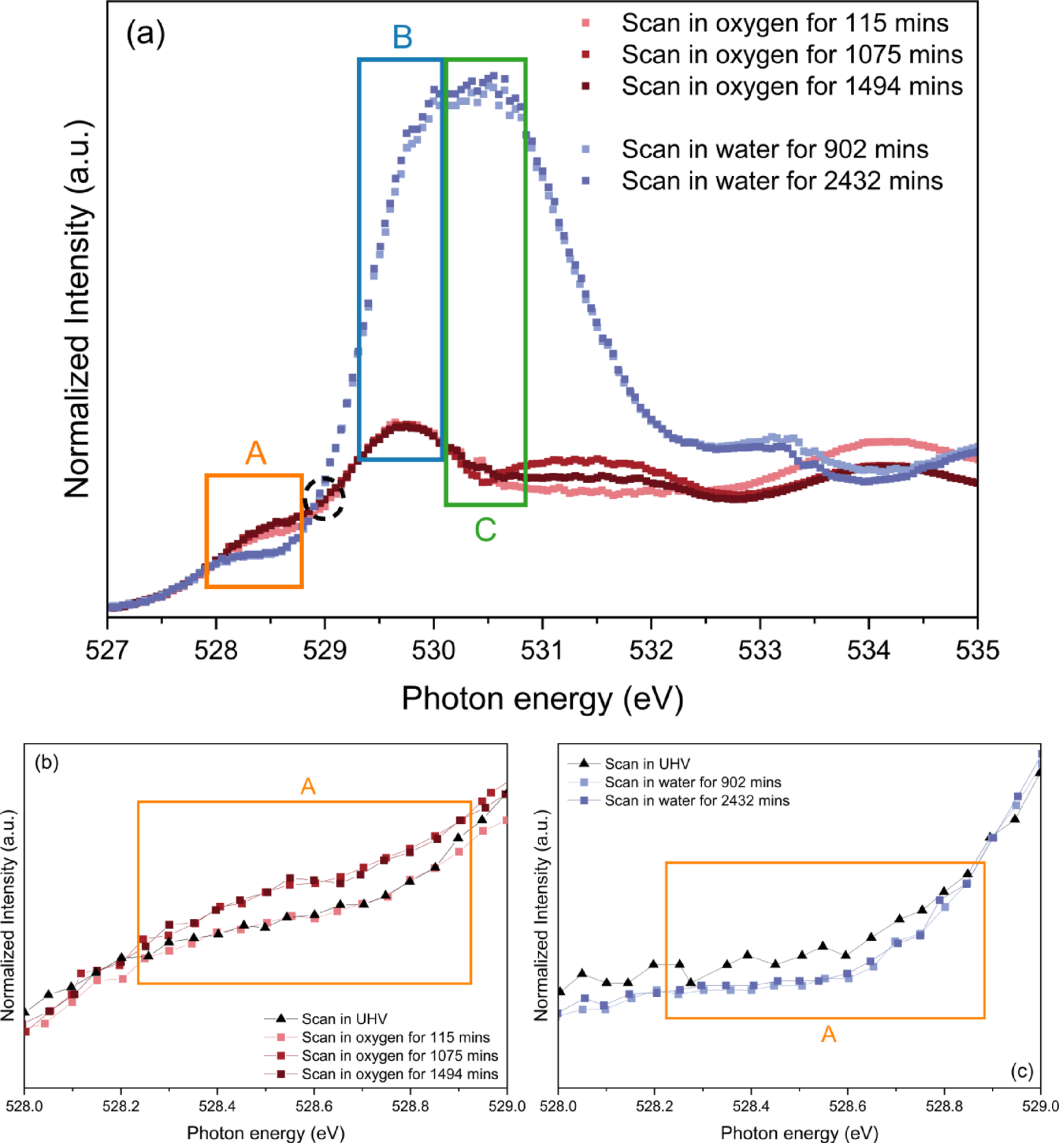
(a) Comparison of the normalized *in situ* O *K*-edge spectra collected on the two LSCrF8255 samples as a function of time under 1 mbar of oxygen and water vapor, respectively. The three scans in oxygen were carried out 115, 1075, and 1494 min after the gas introduction. The two scans in water vapor were carried out 902 and 2432 min after the gas introduction. The isosbestic points are circled. The presence of the isosbestic point, at around 528.9 eV, is in good agreement with previous studies.^12,13^ (b) Comparison of the normalized *in situ* O *K*-edge pre-edge regions measured on the LSCrF8255 sample as a function of time under ultrahigh vacuum (UHV), and subsequently under 1 mbar of oxygen. (c) Comparison of the normalized *in situ* O *K*-edge pre-edge regions measured on the LSCrF8255 sample as a function of time under UHV, and subsequently under 1 mbar of water vapor.

Figure 1 illustrates the O *K*-edge AP-NEXAFS spectra collected as a function of time on two LSCrF8255 samples under ultrahigh vacuum (UHV), followed by 1 mbar of oxygen and water vapor, respectively. In Figure 1, the O *K*-edge spectra were normalized to the isosbestic point at approximately 528.9 eV.^12,13^ The gas-phase oxygen contribution to the O *K*-edge spectra, illustrated in Figure S2 in the Supporting Information, was removed by dividing the electron yield spectra by the transmission spectra collected on the gold foil. Notably, the influence of gas absorption is small in the pre-edge regions (photon energy <530 eV) where the spectral features “A” are highlighted. The pre-edge features “A” correspond to the unoccupied e_g_ ↑ states of Fe/Cr 3d–O 2p mixed character formed due to the O 2p states hybridized with the Fe/Cr 3d states in the octahedral crystal field.^12,54,61^ In Figure 1a, the intensities of the e_g_ ↑ peaks were found higher in dry oxygen than in water vapor. Figure 1b also indicates that the signal of the unoccupied e_g_ ↑ state increases in intensity in oxygen gas flow compared to that measured in UHV prior to the gas introduction. This observation is consistent with an oxidation of a perovskite in dry oxygen and a lowering of the Fermi level corresponding to the depopulation of electronic states near the Fermi level accompanied by annihilation of oxygen vacancies indicated by eq 1.^12,13^ It is worth noting that the e_g_ ↑ peak intensity of scan 1 in oxygen is lower than the following two, suggesting that the surface chemical equilibrium had not been reached 115 min after introducing the oxygen gas flow. The e_g_ ↑ peak intensities of scans 2 and 3 in oxygen, which were obtained 1075 and 1494 min after the gas introduction, are almost identical. On the other hand, Figure 1a,c shows the intensity of feature “A” lower in water vapor as compared to the spectra collected in dry oxygen and the spectrum collected in UHV. The result provides direct experimental evidence showing a relatively higher surface oxygen vacancy concentration on the sample measured in 1 mbar of water vapor, a condition with lower pO_2_, than that in 1 mbar of oxygen, a condition with higher pO_2_. It is conceivable that the oxygen surface exchange kinetics could be enhanced due to the higher surface oxygen vacancy concentration. Our previous study^16^ has demonstrated a 0.7 eV decrease in the activation energy for water surface exchange compared to oxygen surface exchange, and an enhancement of 2 orders of magnitude in the surface exchange kinetics in water vapor (pO_2_ < 1 mbar, pH_2_O = 30 mbar) compared to dry oxygen (pO_2_ = 200 mbar) from 600 to 900 °C. In addition, features “B” and “C” in Figure 1a could be assigned to the unoccupied t_2g_ ↓ and e_g_ ↓ states, respectively.^12,54,61^ The higher 2_2g_ ↓ peak intensities observed in water vapor may be attributed to the change in the covalency.^12^ It could be due to the changes in ligands and the oxidation of oxygen ligands in dry oxygen, which will be discussed in more detail later in this section. Since the energetically higher e_g_ ↓ states are convoluted with the gas absorption peak, the intensity evolution cannot be identified and will not be discussed further.

**Figure 2:**
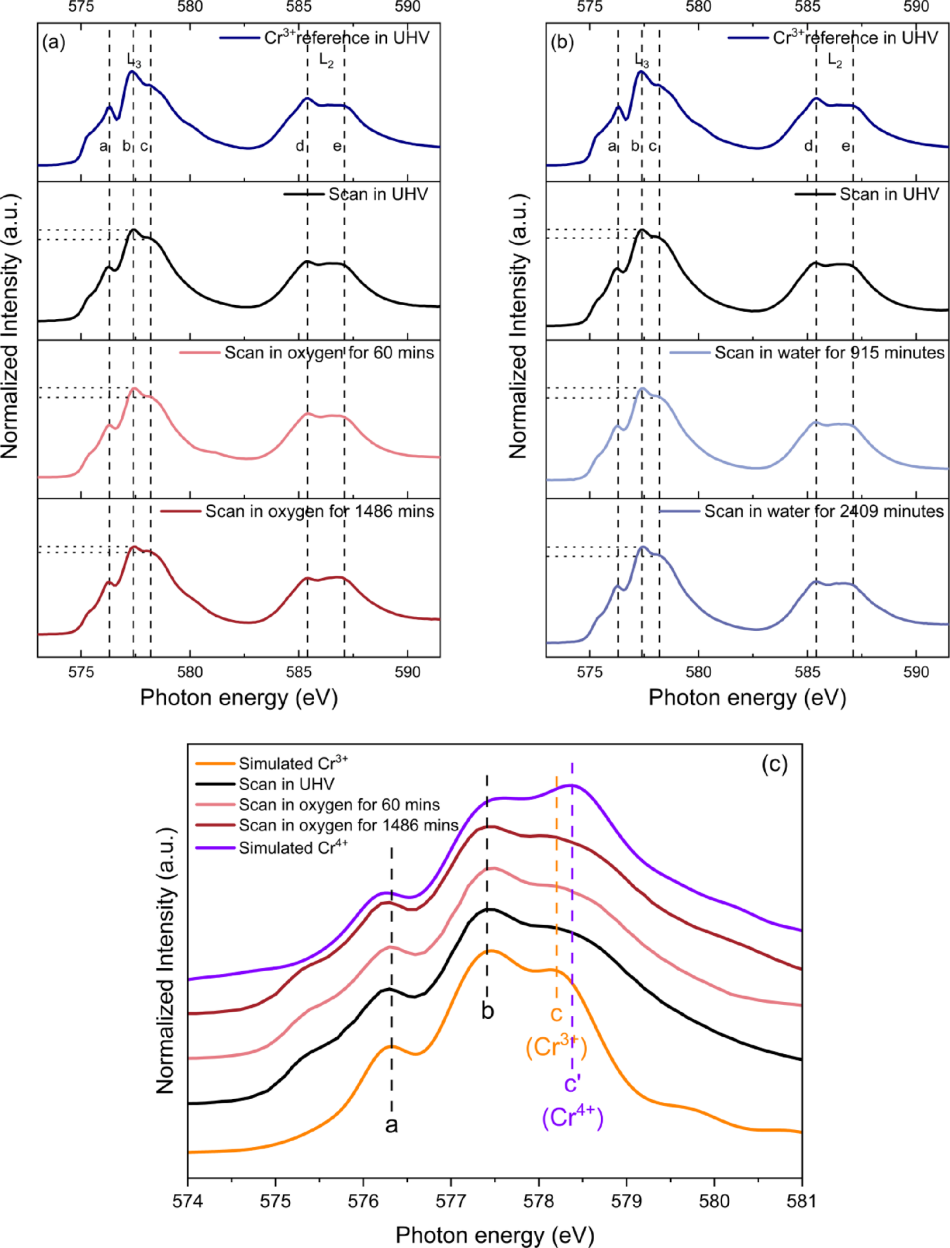
(a, b) The Cr *L*_3,2_-edge AP-NEXAFS spectra collected on the two LSCrF8255 ceramics in UHV and 1 mbar of (a) oxygen and (b) water vapor, respectively, as a function of time. The Cr *L*-edge spectrum collected on the Cr(^3+^)Ox powder with 99.9% purity in UHV is also included as a reference. The two scans in oxygen and water vapor were conducted 60 and 1486 min and 915 and 2409 min after the gas introduction. (c) Comparison of the theoretically simulated Cr *L*-edge spectra for octahedrally coordinated Cr^3+^ (bottom) and Cr^4+^ cations (top) and the experimental data.

Turning the attention toward the valence states of B-site TM ions, the Cr *L*-edge AP-NEXAFS spectra collected as a function of time in UHV and the two gas environments are illustrated in Figure 2.

In Figure 2a,b, the presence of Cr^3+^ species can be confirmed through comparing the Cr *L*-edge spectra of LSCrF to the reference spectrum. The positions of the Cr *L*_3_ edge, guided by the features marked as “a”, “b”, and “c”, and the Cr *L*_2_ edge, guided by the features marked as “d” and “e”, are within 0.1 eV, which is within the margin of repeatability of the beamline monochromator. Notably, as highlighted by the horizontal dotted line, Figure 2a shows minor variations in the intensity ratio between the feature “b” and “c” on the spectra measured in dry oxygen compared to the spectrum in UHV. In contrast, the ratio remains almost constant in water vapor in Figure 2b. Figure 2c further indicates that the changes were likely due to the presence of Cr^4+^ species, described by the spectral feature “c”, by comparing the experimentally derived spectra to the theoretically simulated Cr *L*-edge spectra for octahedrally coordinated Cr^3+^ and Cr^4+^ cations. This finding is consistent with previously reported spectra measured on La_0.75_Sr_0.25_Cr_0.9_Fe_0.1_O_3_ in 3.5 mbar of oxygen at 300 °C, which showed the presence of Cr^4+48^. It deviates from the spectra for Cr^6+^ found on La_0.75_Sr_0.25_Cr_0.9_Fe_0.1_O_3_ in 0.5 mbar of oxygen at 890 °C.^62^ The indication of existing Cr^4+^ in the lattice was not surprising and can be correlated to the oxidation of Cr^3+^ in oxidizing conditions, as described in eq 2:
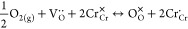
2where Cr_Cr_^×^ denotes Cr^3+^ on Cr^3+^ sites, O_O_^×^ is a neutral lattice oxygen, and Cr_Cr_^·^ represents Cr^4+^ on Cr^3+^ sites.

To deduce the relative amount of the Cr^3+^ and Cr^4+^, the experimentally derived Cr *L*-edge spectra were fitted with the sum of the theoretical curves of octahedral Cr^3+^ and Cr^4+^ cations. The result is displayed in Table 1.

**Table 1:** Atomic Ratio of [Cr^3+^]:[Cr^4+^] Derived from Fitting the Experimental Cr *L*-Edge Spectra with the Sum of the Theoretical Spectra for Octahedrally Coordinated Cr^3+^ and Cr^4+^ Cations for the Sample Measured in UHV and Dry Oxygen Gas Flow

Cr *L*-edge spectra	[Cr^3+^]:[Cr^4+^]
scan in UHV	80:20
scan in oxygen for 60 min	80:20
scan in oxygen for 1486 min	74:26

Table 1 indicates that the presence of Cr^4+^ in the lattice is approximately 20 at %. Despite a subtle increase in the amount found in dry oxygen (within 6 at %), the possibility of Cr oxidization cannot be ruled out. It is also worth noting that the quantification results displayed in Table 1 serve as a basis for comparison since the parameters applied for simulation, the charge transfer energy value Δ, and the crystal field splitting value 10D_q_ were determined by a comparison between simulated and experimentally derived spectra and not by *ab initio* calculations.^48,63^ The same method was employed to deduce the relative ratios of [Cr^3+^]:[Cr^4+^] on the sample measured in water vapor. This result is presented in Table S2 in the Supporting Information and was found to be consistently around 80:20.

**Figure 3:**
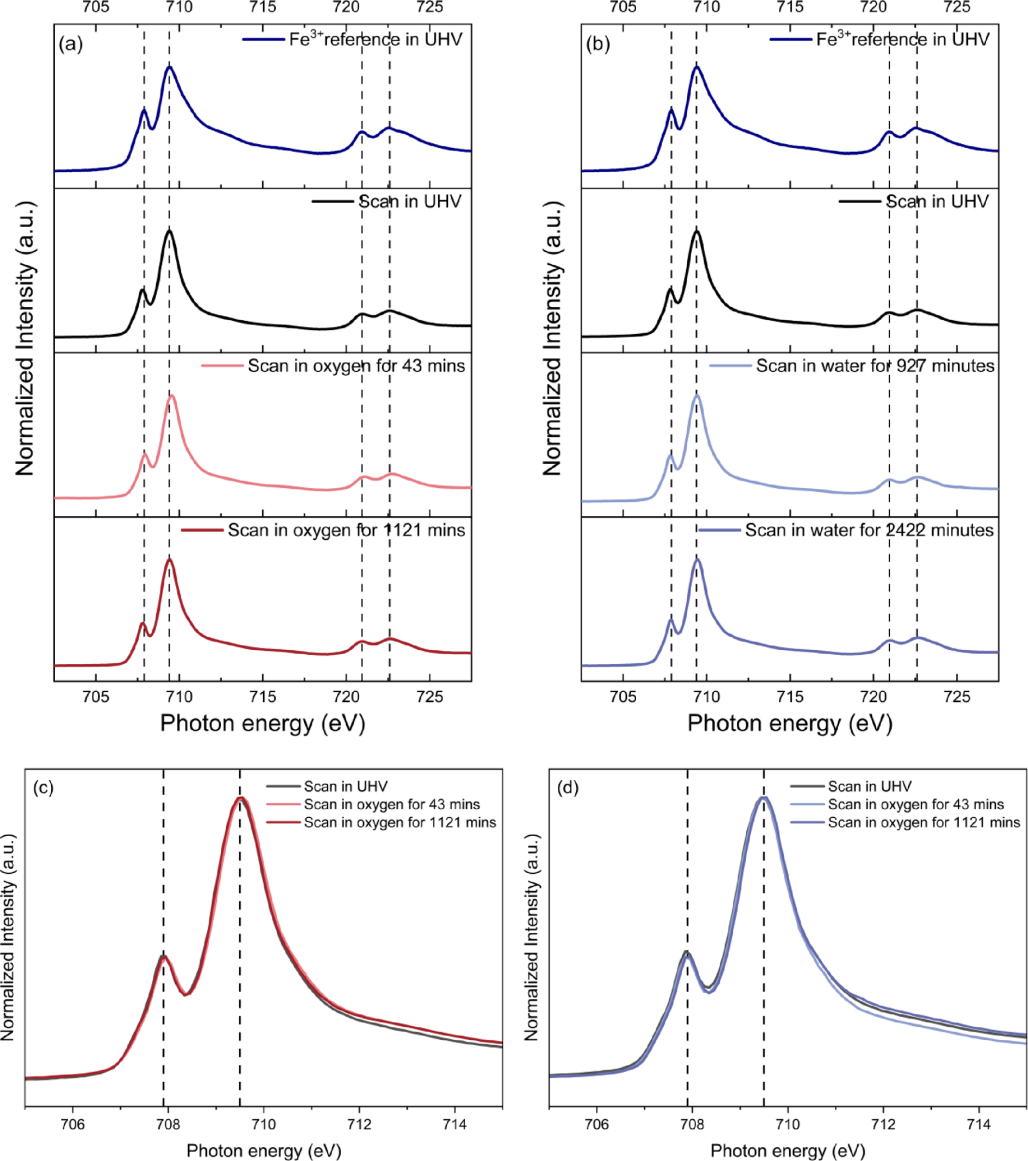
(a, b) The Fe *L*_3,2_-edge AP-NEXAFS spectra collected over time on the two LSCrF8255 ceramics in UHV and 1 mbar of (a) oxygen and (b) water vapor, respectively. The Fe *L*-edge spectrum collected on the Fe(^3+^)Ox powder with 99.9% purity in UHV is also included as a reference. The two scans in oxygen and water vapor were conducted 43 and 1121 min, and 927 and 2422 min after the gas introduction. (c, d) The overlaid Fe *L*_3_-edge spectra of the two samples in UHV and 1 mbar of (c) oxygen and (d) water vapor.

Figure 3 illustrates the Fe *L*-edge AP-NEXAFS spectra collected as a function of time in UHV, and 1 mbar of dry oxygen and water vapor.

In Figure 3, as indicated by the dashed lines, the positions of the Fe *L*_3_ and *L*_2_ edges remained almost identical within the reproducibility of the photon energies of 0.1 eV. The lattice Fe can be assigned to be the “+3” valence state in LSCrF. Additionally, the overlaid Fe *L*_3_-edge spectra in Figure 3c,d show that the intensity ratio of the two *L*_3_ features, the fingerprint for different iron oxidation states,^13,64^ remains consistent with a minor 3% increase found in the scan in UHV in Figure 3d. The constant peak position and the peak ratio indicate the invariable valence state of the lattice Fe.

**Figure 4:**
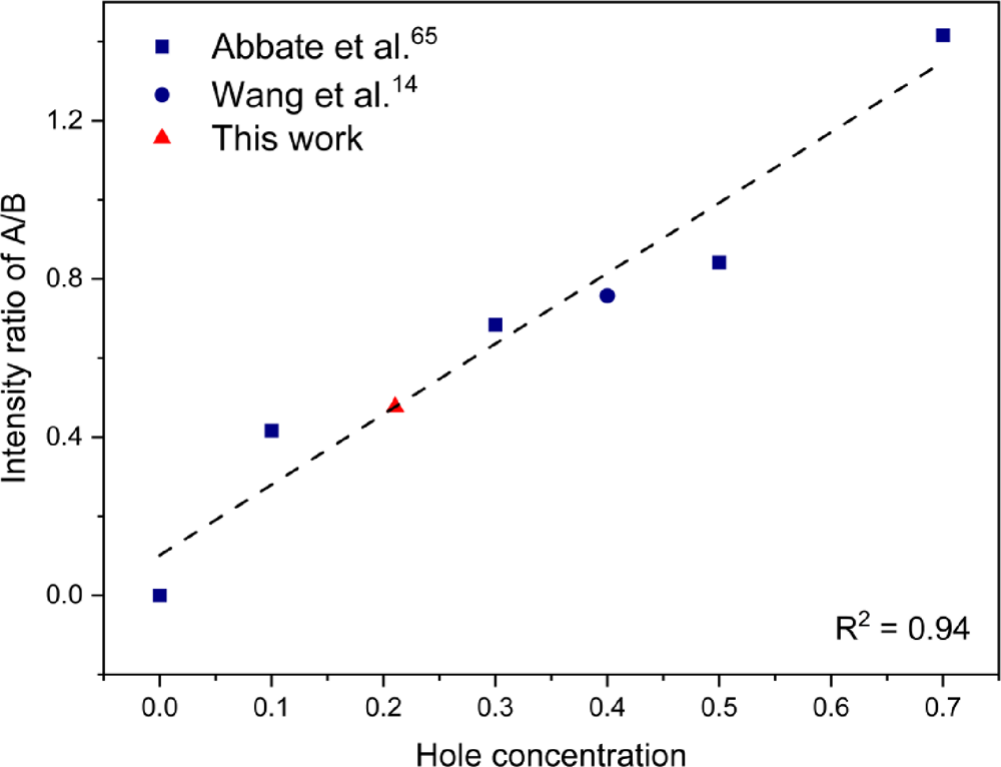
[O_O_^·^] calibration based on the intensity ratio of the feature “A” and “B” on the O *K*-edge spectra, as shown in Figure 1, for the lanthanum strontium TM perovskite oxides as a function of nominal hole concentration. The data represented by blue squares was taken from Abbate et al.^61^ for La_1–*x*_Sr_*x*_FeO_3_ (*x* = 0–0.7), and the data represented by blue dot was taken from Wang et al.^13^ for La_0.6_Sr_0.4_FeO_3_. The dash line indicates the best linear fit.

Defect models for LSCrF can be proposed through surveying the valence states in 1 mbar of dry oxygen and water vapor. First, in dry oxygen, the O_2_ incorporation and evolution reaction can be expressed using eq 3, in which a surface lattice oxygen is oxidized:
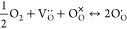
3where O_O_^·^ denotes a single positively charged oxygen ion. Alternatively, the surface Cr^3+^ could be oxidized to Cr^4+^ (Cr_Cr_^·^), as described in eq 2. To quantify the concentration of O_O_^·^, the intensity ratio of the features “A” and “B” in the O *K*-edge spectra shown in Figure 1 can be used as a descriptor based on a linear relationship.^13^ The calibration for the [O_O_^·^] is illustrated in Figure 4.

Since Figure 1 shows the intensity ratio of A/B plateaued between the scans carried out in oxygen after 1075 and 1494 min after the gas introduction, the [O] is assumed saturated. The saturated [O_O_^·^] was estimated to be 0.2 ± 0.1, indicated by the red triangle in Figure 4, in (La_0.8_Sr_0.2_)_0.95_Cr_0.5_Fe_0.5_O_3-δ_ equilibrated in 1 mbar of oxygen. The [O_O_^·^] in the sample prior to the gas introduction was estimated through the scan in UHV and was found negligible. The significant 20 at % increase in [O_O_^·^] in dry oxygen, compared to the 6 at % increase in [Cr_Cr_^·^], indicates that electron holes in LSCrF are mainly localized on the lattice oxygen rather than the TM cations. The *in situ* results further revealed that, contrary to the traditional view that the B-site TMs are the dominant redox-active species for LSCrF,^3^ the oxygen anions in the near surface were proven to be a significant redox partner to the molecular oxygen due to the hybridization of Fe/Cr 3d and O 2p in the octahedral crystal field. As described by eq 3, during the oxygen incorporation in dry oxygen, the lattice oxygen was oxidized while the oxygen adsorbate was reduced. In addition, under 1 mbar water vapor, the O *K*-edge spectra in Figure 1 also suggest the formation of electronic states near the Fermi level, accompanied by creation of oxygen vacancies as described by eq 1. In contrast, the oxidation states of Fe and Cr remained constant. More details regarding the water surface exchange mechanism are revealed by O 1s AP-XPS spectra in the section below.

### 3.2 Oxygen Adsorbates in O_2_ and H_2_O

**Figure 5:**
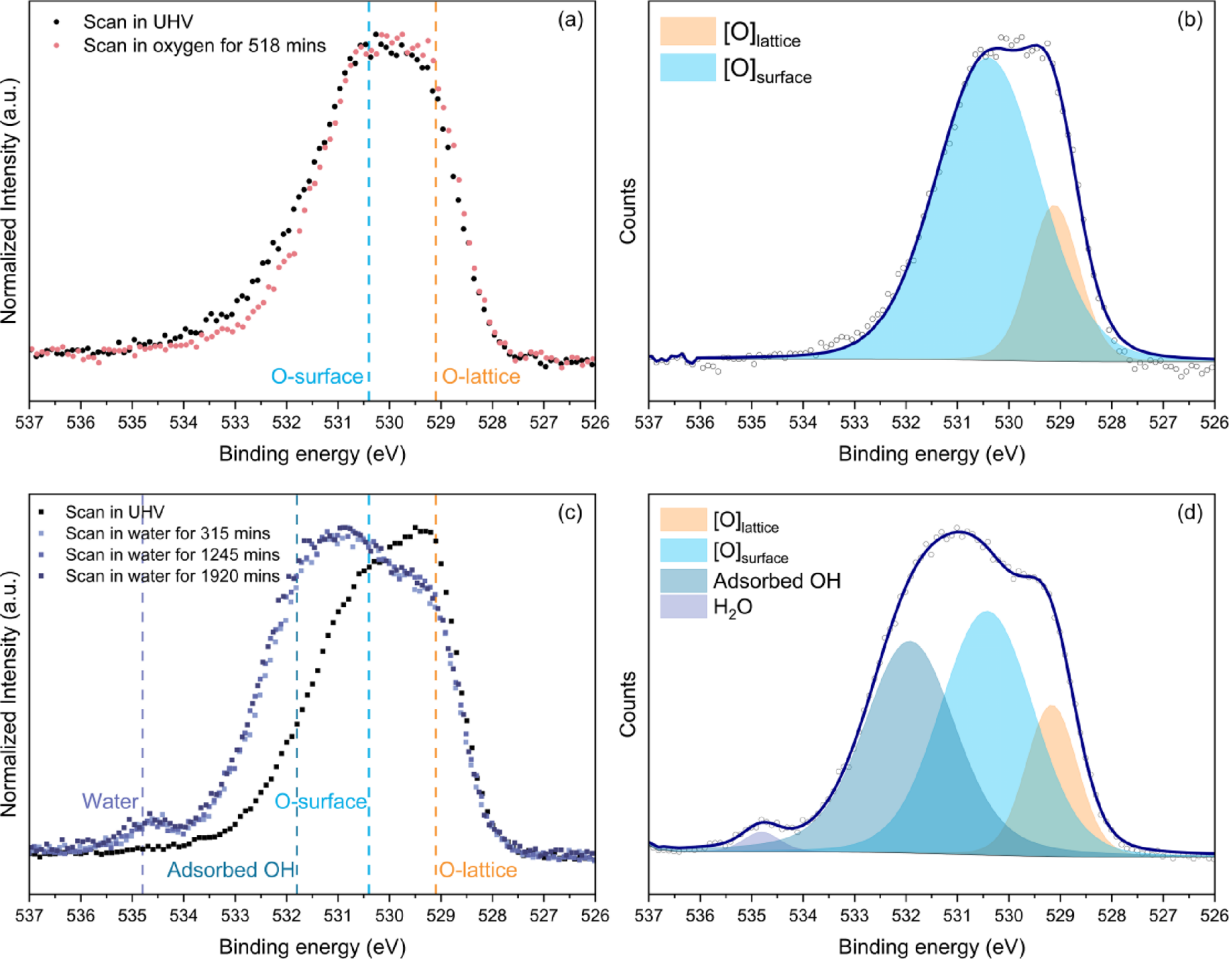
(a) O 1s AP-XPS spectra measured on the LSCrF8255 sample with hν = 720 eV in UHV, and 518 min after introducing oxygen gas flow. (b) The fitting of the O 1s spectrum collected in dry oxygen. (c) O 1s AP-XPS spectra measured on the LSCrF8255 sample with hν = 720 eV in UHV, and 315, 1245, and 1920 min after introducing water vapor gas flow. (d) The fitting of the O 1s spectrum collected in water vapor 1245 min after the gas introduction.

Figure 5 illustrates the O 1s AP-XPS spectra collected as a function of time on the two LSCrF8255 samples under UHV, followed by introduction of 1 mbar of oxygen and water vapor, respectively.

In Figure 5a, the O 1s spectral shapes obtained in UHV and in dry oxygen are similar. The peak deconvolution indicates that the O 1s can be fitted by two components, as illustrated in Figure 5b. The “lattice” component, at a BE of 529.1 eV, corresponds to the oxygen species in a perovskite lattice [O]_lattice_,^44,48,65,66^ while the “surface” component, at a BE of 530.4 eV, represents the oxygen species in surface layers [O]_surface_.^44,48^ The fitting results are presented in Table S3 in the SI, demonstrating a consistent ratio of [O]_surface_:[O]_lattice_. Figure 5c,d shows two additional components in the O 1s spectra collected in water vapor, as compared to the spectra in UHV and dry oxygen. The component at 531.8 eV is related to adsorbed hydroxyl groups,^48^ and the component at 534.8 eV is associated with adsorbed water molecules^67^ on the LSCrF surface. The fitting results are displayed in Table S4 in the Supporting Information. In Table S4, a consistent ratio of [O]_surface_:[O]_lattice_ (2.90 ± 0.05) was also found. In particular, there was a large extent of adsorbed hydroxyl group (approximately 30 at %) formed on the LSCrF8255 surface. The finding is in contrast to the study on La_0.75_Sr_0.25_Cr_0.9_Fe_0.1_O_3_ measured in 3.5 mbar of the H_2_O and H_2_O/H_2_ mixture,^48^ where no adsorbed oxygen species were detected. The observed hydroxyl groups provide strong evidence for the water surface exchange reaction, which was studied previously by us^16^ and by Nenning et al.^14^ using the isotopic exchange depth profiling (IEDP) technique. In water vapor, the adsorption and dissociation of H_2_O in the gas phase onto the perovskite surface is likely to occur on oxygen vacancy sites, forming two hydroxyl groups with an adjacent lattice oxygen as described in eq 4:
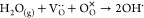
4

Furthermore, O incorporation could occur through the desorption of H_2_O, described in eq 5:
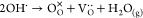
5

It can be deduced from eqs 4 and 5 that the nonredox oxygen exchange occurs in water vapor, in contrast to the ORR occurring in dry oxygen (eq 1). The mechanistic differences in surface exchange also involve the formation of hydrogen bonds. These differences, along with a relatively higher surface oxygen vacancy concentration, indicated in Figure 1, can lead to a significant enhanced water surface exchange kinetics as compared to that of dioxygen.^16^ Finally, the concentration of the adsorbed water molecules was found consistent at 1 at % on the sample surface in the water vapor flow.

### 3.3 Surface Composition Evolution in O_2_ and H_2_O

**Figure 6:**
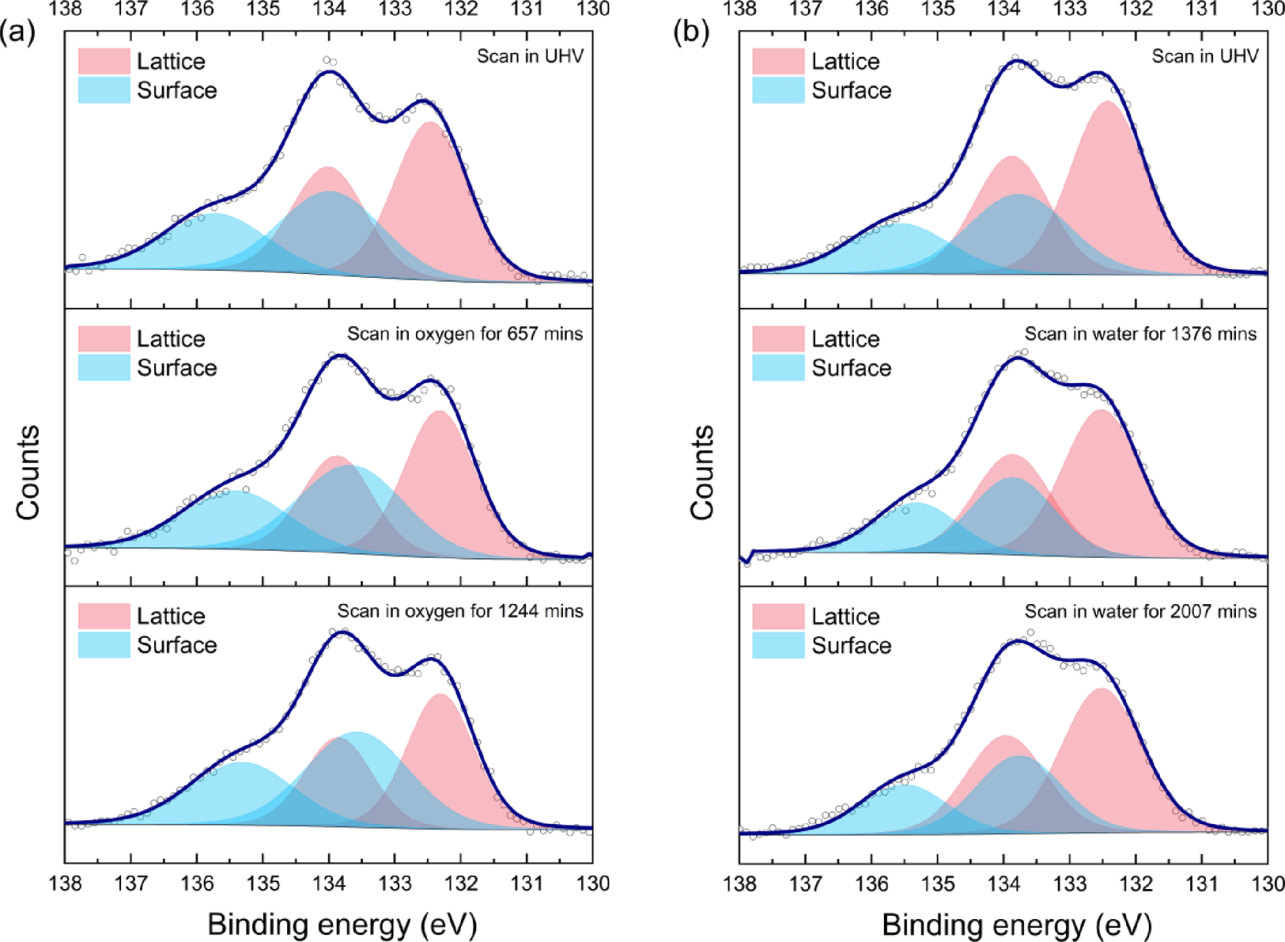
(a, b) Sr 3d AP-XPS spectra measured on two LSCrF samples with *h*ν = 720 eV in UHV, followed by 1 mbar of (a) dry oxygen and (b) water vapor, respectively.

More detailed assessment on the surface chemistry evolution under the two gas environments was provided by AP-XPS. Figure 6 presents the Sr 3d AP-XPS spectra collected over time on the two LSCrF8255 samples under UHV, and then under 1 mbar of oxygen and water vapor, respectively.

**Figure 7:**
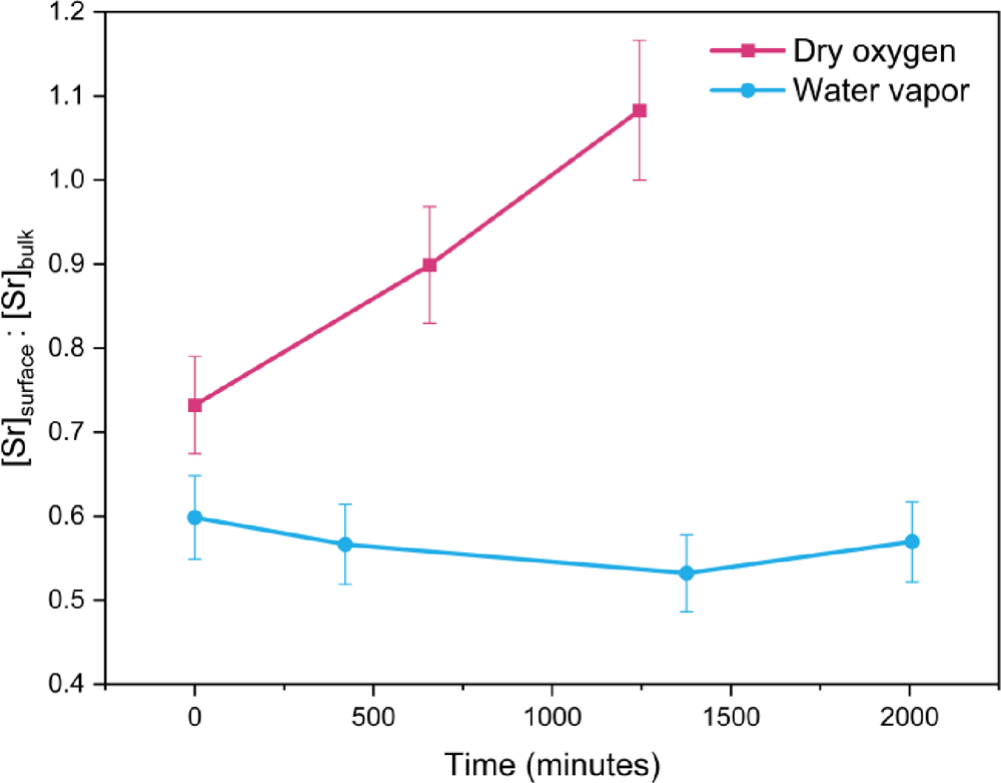
Atomic ratio of [Sr]_surface_:[Sr]_lattice_ obtained as a function of time after the gas introduction of the samples measured in dry oxygen and water vapor. The initial values for each sample were measured in UHV at *t* = 0 min.

In Figure 6, the deconvolution of Sr 3d spectra shows two sets of spin–orbit split doublets, indicating the presence of two different chemical environments for Sr. The doublet with a lower BE aries from the Sr in the perovskite lattice in the near-surface region ([Sr]_lattice_), while the doublet with a higher BE corresponds to the Sr in surface species ([Sr]_surface_). The atomic ratio of [Sr]_surface_:[Sr]_lattice_ is an important indicator of the extent of Sr segregation, and the values obtained from peak fitting are displayed in Figure 7.

In Figure 7, an increase in the atomic ratio of [Sr]_surface_:[Sr]_lattice_ was observed for the sample measured in dry oxygen, indicating that the amount of Sr surface species was increasing over time. Counterintuitively, it was found that the ratio remained approximately constant for the sample measured in water vapor. As indicated by the O *K*-edge spectra in Figure 1, there was a higher level of surface oxygen vacancies on the sample exposed to water vapor. Thus, the electrostatic driving force for Sr segregation, generally accepted to be between the V_O_^··^ and Sr_La_^′^, would be greater, which in turn should result in a higher [Sr]_surface_.^27^ However, previous discussion has revealed the presence of a substantial quantity of electron holes ([O_O_^·^] and [Cr_Cr_^·^]) on the sample surface in dry oxygen, which could serve as an additional driving force for Sr segregation. Furthermore, the fewer Sr surface species found in water vapor, as compared to dry oxygen, could be due to Sr(OH)_2_ being the most volatile Sr species.^68^ The observed changes in the Sr surface contents are very likely due to a combination of Sr surface segregation and vapor-phase transport under a gas flow at a surface temperature of 500 °C. As a result of these processes, the volatile Sr(OH)_2_ formed on the sample surface could be carried away by the water vapor flow. Additionally, it is worth noting that the La chemical bonding environments are consistent in both of the atmospheres. Figure S3 in the Supporting Information illustrates the La 3d AP-XPS spectra obtained over time on the two samples, first under UHV and then under dry oxygen and water vapor, respectively. In Figure S3, the magnitude of the multiplet splitting was found to be 4.3 eV for both samples, which is in good agreement with the literature value for La_2_O_3_ and lanthanum transition metal perovskite oxides.^27,69−72^

**Figure 8:**
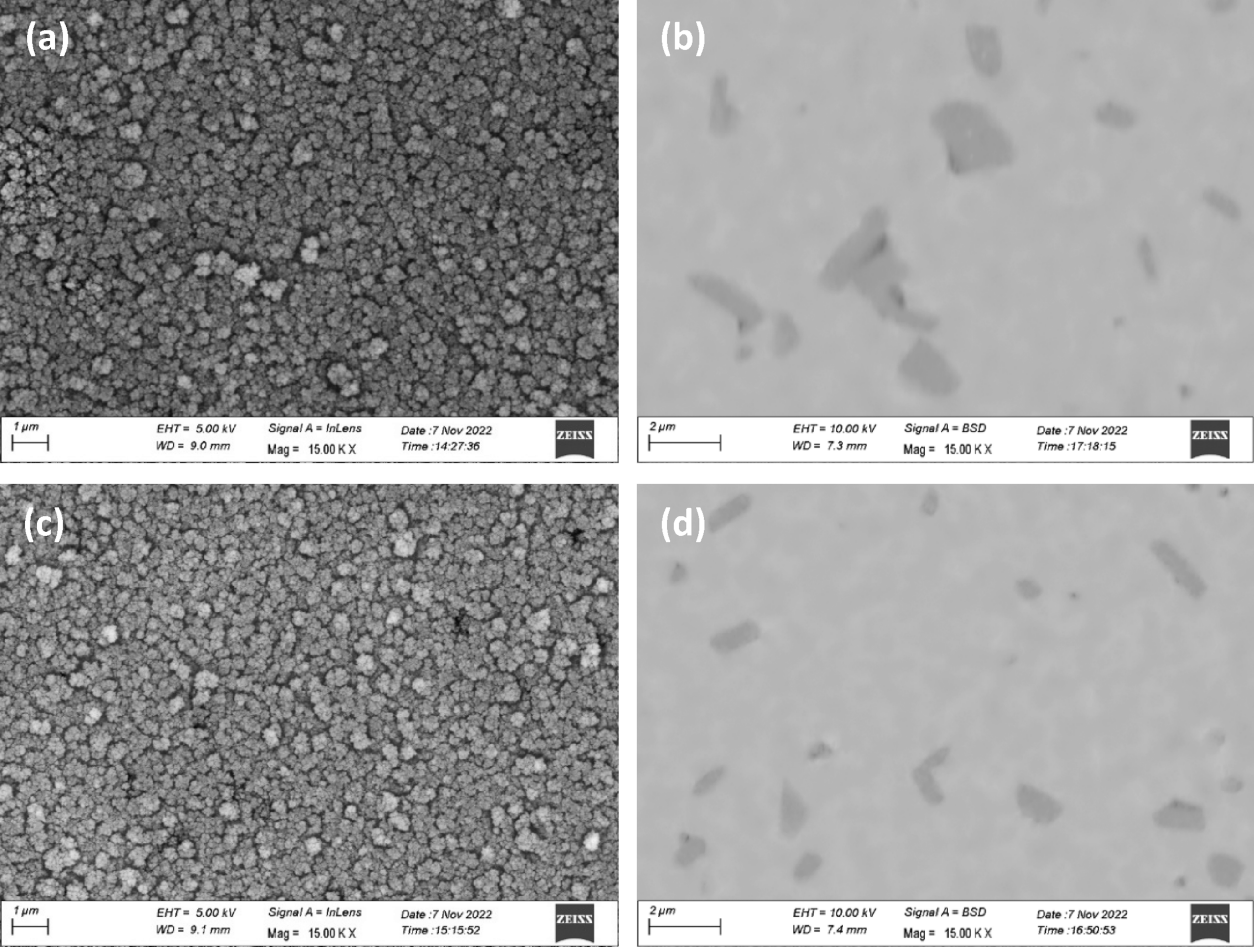
(a) Secondary electron (SE) and (b) backscattered electron (BSE) images of the sample measured in dry oxygen, and (c) SE and (d) BSE images of the sample measured in water vapor.

In addition to the Sr surface segregation, a phase separation was also observed. The SEM micrographs of the two post-tested samples are illustrated in Figure 8.

Figure 8a,c shows a nanostructured LSCrF, with a grain size of a few hundred nanometers. The formation of a secondary phase, which appears darker in the BSE image of Figure 8b,d, was confirmed to occur during the AP-XPS and AP-NEXAFS measurements when the sample was exposed to the reactive gases, as no secondary phase was detectable on the as-sintered samples.^15^ The chemical composition of the secondary phase was determined through energy-dispersive X-ray spectroscopy (EDX) in SEM mode (SEM-EDX), revealing that it was deficient in A-site cations, markedly in La and to a lesser extent in Sr, and enriched in B-site cations, markedly in Fe and to a lesser extent in Cr. The SEM-EDX maps obtained from the two post-tested samples are illustrated in Figure S4 in the Supporting Information. This finding is in line with the LSCrF samples annealed in both of the dry oxygen and water vapor environments reported in our previous studies.^15,16,27^ The result suggests that the material is transforming from A-site-deficient A_0.95_BO_3-δ_ to an ABO_3-δ_ perovskite oxide during the *in situ* measurement. The XRD patterns of those two samples are illustrated in Figure S5 in the Supporting Information. In Figure S5a, all the diffraction peaks are consistent in position. The structure refinement displayed in Figure S5B further indicates that the main phase has an orthorhombic crystal structure with space group *Pnma*. The result agrees well with the as-sintered LSCrF8255.^15^ As highlighted on the figure, the diffraction peak at 2θ = 29.3, 30.5, 34.3 and 34.4° could be due to the formation of the secondary phase.

## 4 Conclusions

In this study, AP-XPS and AP-NEXAFS were applied to investigate the surface defect equilibria and surface composition evolution of LSCrF8255, a model MIEC perovskite oxide. The measurements were performed under UHV, and 1 mbar of molecular oxygen and water vapor at a surface temperature of 500 °C. The *in situ* O *K*-edge, Cr *L*-edge, and Fe *L*-edge AP-NEXAFS spectra indicated that the surface lattice oxygen was the key redox-active species under both gas environments. In dry oxygen, the oxygen exchange was charge compensated by electron holes. A 20 at % increase in [O_O_^•^] was demonstrated, compared to a 6 at % increase in [Cr_Cr_^•^], showing that the electron holes are primarily located on the oxygen sites. In water vapor, a relatively higher concentration of oxygen vacancy was observed. The evidence suggests that, in contrast to the conventional view that the TM cations are the dominant redox partner to oxygen adsorbates, the TM-O_6_ octahedron as a whole should be regarded as the redox-active entity. The *in situ* O 1s AP-XPS spectra further revealed that the adsorption and dissociation of H_2_O onto the perovskite surface were through the formation of hydroxyl groups. These findings show that distinct mechanisms underlie water surface exchange compared to that of dioxygen, including nonredox processes where charge transfer is not required, as well as the formation of hydrogen bonds. These factors combined with a higher oxygen vacancy concentration could potentially facilitate the exchange kinetics in the water vapor.

In terms of surface composition evolution, the Sr 3d AP-XPS spectra showed that the amount of Sr surface species increased over time in dry oxygen due to Sr surface segregation, with the presence of oxygen holes on the surface acting as an additional driving force. In contrast, the amount remained roughly constant for the sample measured in water vapor. The less Sr contents observed on the sample in water vapor could be due to the volatility of Sr(OH)_2_. In addition, *ex situ* SEM and XRD analyses indicated the occurrence of phase separation. The secondary phase that formed was deficient in A-site cations, particularly in La and relatively in Sr, and enriched in B-site cations, particularly in Fe and relatively in Cr. It suggests that the material transformed from A-site-deficient A_0.95_BO_3-δ_ to an ABO_3-δ_ perovskite oxide during the *in situ* measurement.

## Supporting Information Available

Heating assembly setup for ambient pressure (AP)–X-ray photoelectron spectroscopy (XPS) and near-edge X-ray absorption fine-structure spectroscopy (NEXAFS); O *K*-edge XPS spectra as a function of time; La 3d AP-XPS spectra; SEM-EDX maps of LSCrF samples in dry oxygen, and water vapor; summary of literature values of slater integrals, spin–orbit coupling parameter (SO), difference between core-hole potential parameter and Hubbard *U* value, hopping parameters for *e*_g_ (T(*e*_g_)) and *t*_2g_ (T(*t*_2g_)), crystal field splitting value, and charge transfer energy value (Δ) for simulating the octahedrally coordinated Cr^3+^ and Cr^4+^ Cr *L*-edge spectra; atomic ratio of [Cr^3+^]:[Cr^4+^] derived from Cr *L*-edge spectra for measurement in UHV and water vapor; the atomic ratio of [O]_surface_:[O]_lattice_ derived from fitting the O 1s AP-XPS spectra; and the atomic ratio of different oxygen species derived from fitting O 1s AP-XPS spectra obtained from measurement in UHV and water vapor (PDF)
