# Supplementary material for: Studying Surface Chemistry of Mixed Conducting Perovskite Oxide Electrodes with Synchrotron-Based Soft X-rays
Source: J Phys Chem C Nanomater Interfaces. 2023 Oct 9;127(41):20325–36. doi: 10.1021/acs.jpcc.3c04278 (PMC10591506; doi:10.1021/acs.jpcc.3c04278)
Supplement: Supplementary file 1 — jp3c04278_si_001.pdf [file jp3c04278_si_001.pdf]

Supplementary information

Studying Surface Chemistry of Mixed Conducting Perovskite Oxide Electrodes with Synchrotron-based Soft X-rays

Zijie Sha[a], Gwilherm Kerherve[a], Matthijs A. van Spronsen[b], George E. Wilson[a], John A. Kilner[a], Georg Held[b], and Stephen J. Skinner[a]

[a]Dept. of Materials, Exhibition Road, Imperial College London, London, SW7 2AZ, UK

[b]Diamond Light Source Ltd, Didcot, OX11 0DE, UK

s.skinner@imperial.ac.uk

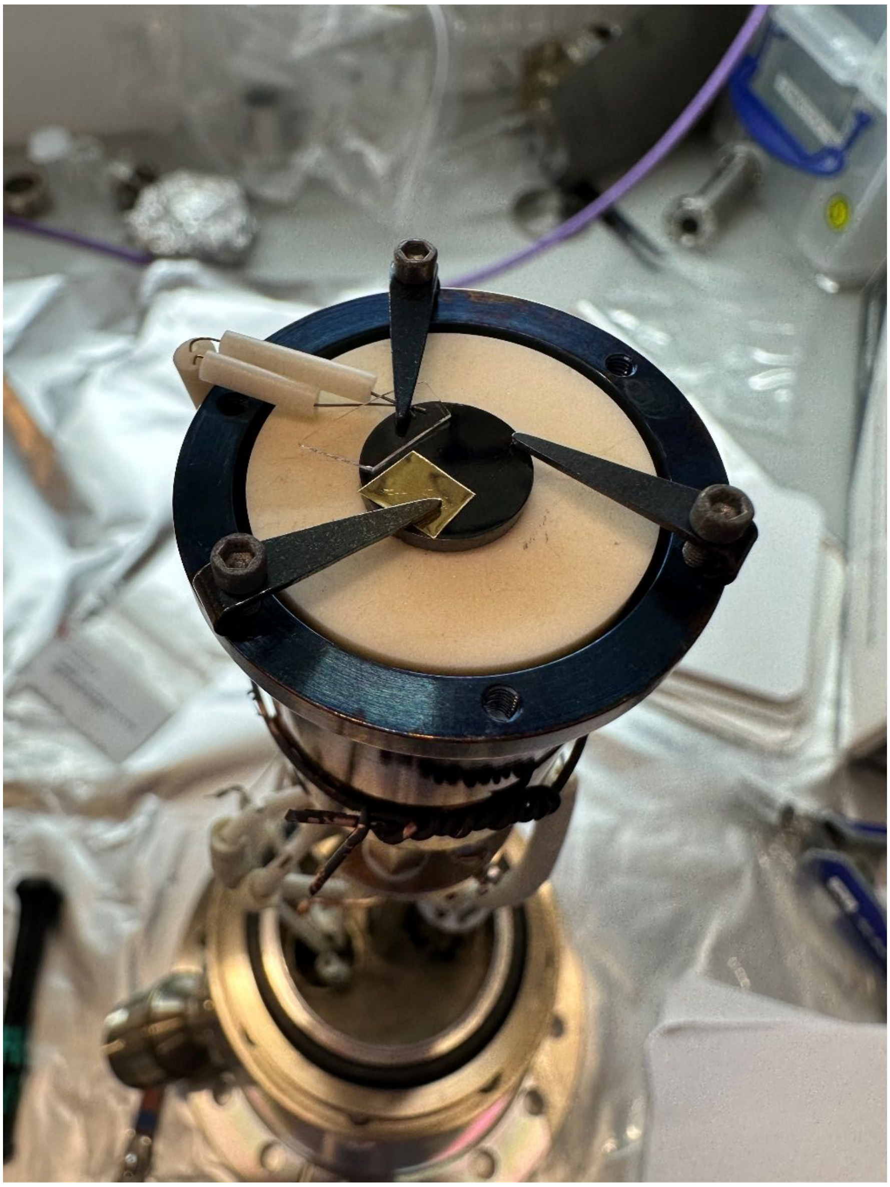

Figure S1. The heating assembly set-up for ambient pressure (AP) – X-ray photoelectron spectroscopy (XPS) and near-edge X-ray absorption fine-structure spectroscopy (NEXAFS).

Table S1. Summary of the literature values[1–3] of the slater integrals (F_{dd}, F_{pd}, and G_{pd}), the spin-orbit coupling parameter (SO), the difference between the core-hole potential parameter and the Hubbard U value (U_{pd}-U_{dd}), the hopping parameters for e_g (T(e_g)) and t_{2g} (T(t_{2g})), the crystal field splitting value (10D_q, which defined as the energy difference between the t_{2g} and the e_g states in octahedral symmetry), and the charge transfer energy value (Δ) for simulating the octahedrally coordinated Cr^{3+} and Cr^{4+} Cr *L*-edge spectra.

**Parameters**	**Cr^{3+}**	**Cr^{4+}**
F_{dd}, F_{pd}, and G_{pd}	0.9	0.9
SO	1.04	0.95
U_{pd}-U_{dd}	1	3
T(e_g)	1.35	1
T(t_{2g})	0.65	0.5
10D_q (eV)	1.2	1.6
Δ	7	1

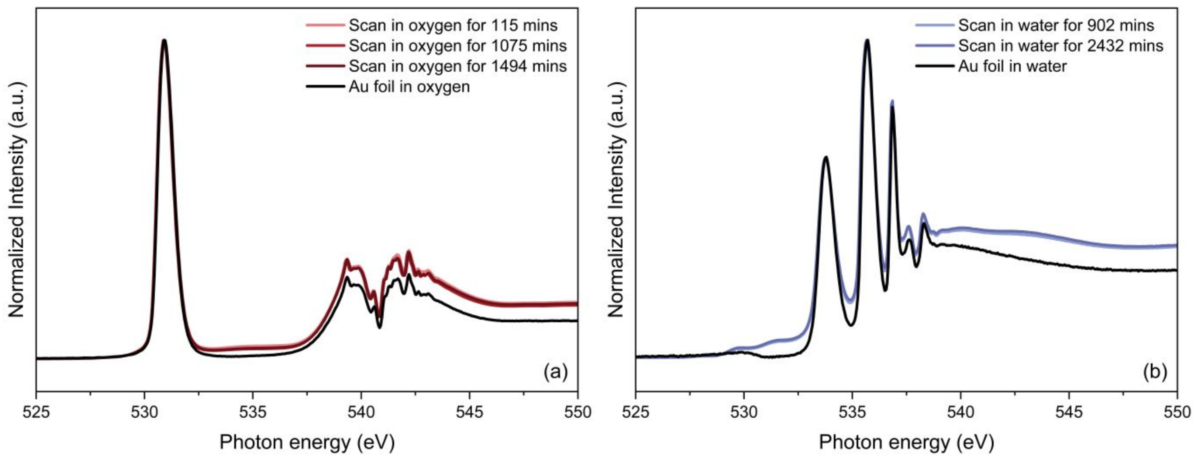

Figure S2. Overlay of the O *K*-edge spectra collected in 1 mbar of (a) oxygen and (b) water vapour as a function of time showing the contribution of gas-phase oxygen.

Table S2. The atomic ratio of [Cr^{3+}] : [Cr^{4+}] derived from fitting the experimental Cr *L*-edge spectra with the sum of the theoretical spectra for octahedrally coordinated Cr^{3+} and Cr^{4+} cations for the sample measured in UHV and water vapour gas flow.

**Cr *L*-edge spectra**	**[Cr^{3+}] : [Cr^{4+}]**
Scan in UHV	80:20
Scan in water vapour for 915 mins	80:20
Scan in water vapour for 2409 mins	79:21

Table S3. The atomic ratio of $[O]_{surface} : [O]_{lattice}$ derived from fitting the O 1*s* AP-XPS spectra obtained from the sample measured in UHV and dry oxygen gas flow.

O 1*s* spectra	$[O]_{surface}$ at.%	$[O]_{lattice}$ at.%
Scan in UHV	81	19
Scan in dry oxygen for 518 mins	80	20

Table S4. The atomic ratio of different oxygen species derived from fitting the O 1*s* AP-XPS spectra obtained from the sample measured in UHV and water vapour gas flow.

O 1*s* spectra	$[O]_{surface}$ at.%	$[O]_{lattice}$ at.%	[OH] at.%	$[H_2O]$ at.%
Scan in UHV	74	26	N/A	N/A
Scan in water vapour for 315 mins	46	16	37	1
Scan in water vapour for 1245 mins	44	15	40	1
Scan in water vapour for 1920 mins	43	15	41	1

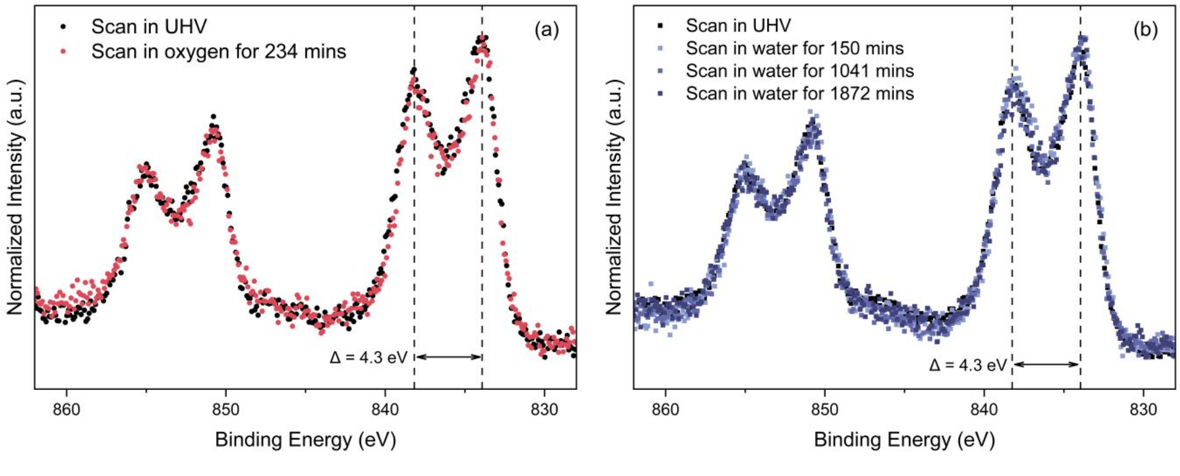

Figure S3. (a – b) La 3*d* AP-XPS spectra measured on two LSCrF samples with *hv* = 1070 eV in UHV, followed by 1 mbar of (a) dry oxygen, and (b) water vapour, respectively.

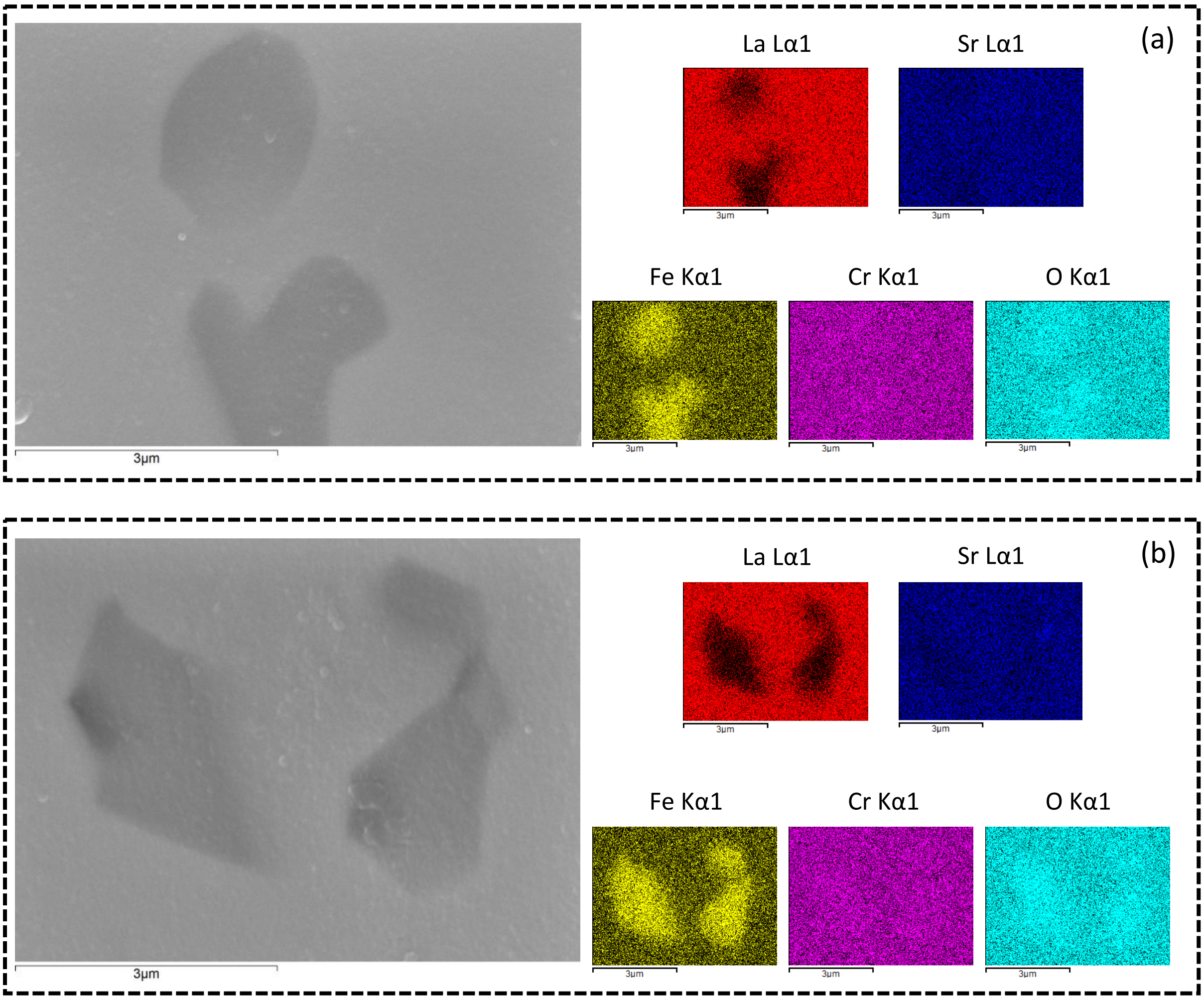

Figure S4. (a – b) The SEM-EDX map of the two LSCrF samples measured in (a) dry oxygen, and (b) water vapour, respectively.

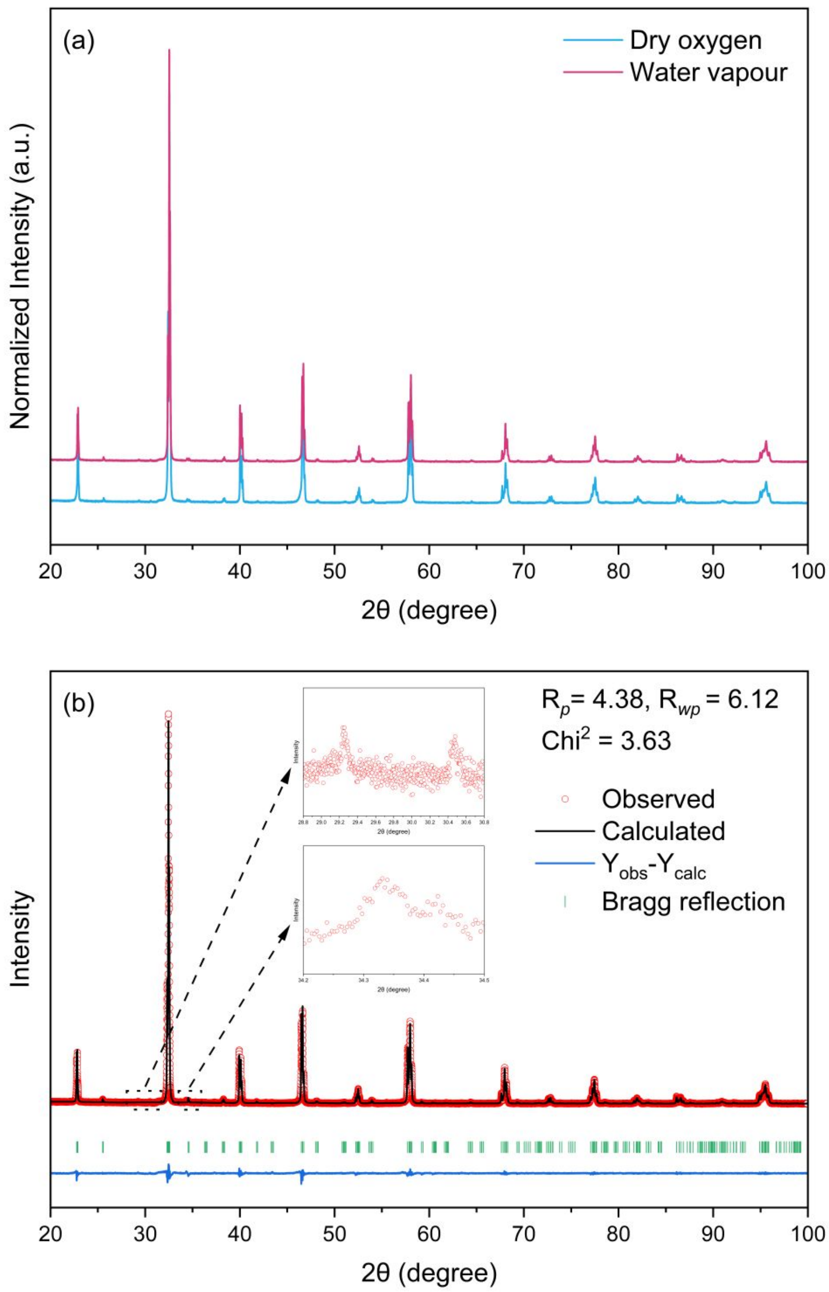

Figure S5. (a) Comparison of the XRD patterns of the two LSCrF samples measured in dry oxygen and water vapour. (b) XRD patterns of the LSCrF sample measured in water vapour fitted by Le Bail refinement[4], highlighting additional diffraction peaks which could be due to the formation of a secondary phase. The red circles are the observed experimental data, the black solid line are the calculated intensities, the blue line is the difference between the observed and calculated data, and the green vertical bars represents the Bragg reflection of the main phase fitted with orthorhombic structure with space group *Pnma*.

References

1. Paloukis, F. *et al.* Insights into the Surface Reactivity of Cermet and Perovskite Electrodes in Oxidizing, Reducing, and Humid Environments. *ACS Appl. Mater. Inter.* **9**, 25265–25277 (2017).

2. Tesch, R. & Kowalski, P. M. Hubbard U parameters for transition metals from first principles. *Phys. Rev. B* **105**, (2022).

3. Shkvarin, A. S., Yablonkikh, M. V., Yarmoshenko, Y. M., Merentsov, A. I., Senkovskiy, B. V., Avila, J., Asensio, M. & Titov, A. N. Electronic structure of octahedrally coordinated Cr in Cr_xTiX_2 (X = Se, Te) and $Ti_xCr_{1-x}Se_2$. *J. Electron Spectrosc. Relat. Phenom.* **206**, 12–17 (2016).

4. Le Bail, A. Whole powder pattern decomposition methods and applications: A retrospection. *Powder Diffr.* **20**, 316–326 (2005).
